# Assembly and Analysis of *Haemonchus contortus* Transcriptome as a Tool for the Knowledge of Ivermectin Resistance Mechanisms

**DOI:** 10.3390/pathogens12030499

**Published:** 2023-03-22

**Authors:** David Emanuel Reyes-Guerrero, Verónica Jiménez-Jacinto, Rogelio Alejandro Alonso-Morales, Miguel Ángel Alonso-Díaz, Jocelyn Maza-Lopez, René Camas-Pereyra, Agustín Olmedo-Juárez, Rosa Isabel Higuera-Piedrahita, María Eugenia López-Arellano

**Affiliations:** 1 Centro Nacional de Investigación Disciplinaria en Salud Animal e Inocuidad, Instituto Nacional de Investigaciones Forestales, Agrícolas y Pecuarias, Carr. Fed. Cuernavaca-Cuautla 8534, Jiutepec C.P. 62574, Morelos, Mexico; reyes.david@inifap.gob.mx (D.E.R.-G.);; 2 Facultad de Medicina Veterinaria y Zootecnia, Universidad Nacional Autónoma de México, Ciudad Universitaria, C.P. 04510, Ciudad de México, Mexico; 3 Unidad Universitaria de Secuenciación Masiva y Bioinformática, Instituto de Biotecnología, Universidad Nacional Autónoma de México, Av. Universidad 2001, Chamilpa, Cuernavaca C.P. 62210, Morelos, Mexico; 4 Centro de Enseñanza, Investigación y Extensión en Ganadería Tropical, Facultad de Medicina Veterinaria y Zootecnia, Universidad Nacional Autónoma de México, Km. 5. Carr. Fed. Tlapacoyan-Martínez de la Torre, Martínez de la Torre C.P. 93600, Veracruz, Mexico; 5 Facultad de Estudios Superiores Cuautitlán, Facultad de Medicina Veterinaria y Zootecnia, Universidad Nacional Autónoma de México, Cuautitlán-Teoloyucan Km 2.5, Col. San Sebastián Xhala. Cuautitlán, C.P. 54714, Estado de México, Mexico

**Keywords:** *Haemonchus contortus*, ivermectin, RNA-Seq, differential expression of genes, functional enrichment

## Abstract

*Haemonchus contortus* (Hc) is an important parasitic nematode of small ruminants. In this study we assembled the transcriptome of Hc as a model to contribute to the knowledge about the profile of the differential gene expression between two Mexican Hc strains under different anthelmintic resistance statuses, one susceptible and the other resistant to ivermectin (IVMs and IVMr, respectively), in order to improve and/or to have new strategies of control and diagnosis. The transcript sequence reads were assembled and annotated. Overall, ~127 Mbp were assembled and distributed into 77,422 transcript sequences, and 4394 transcripts of the de novo transcriptome were matched base on at least one of the following criteria: (1) Phylum Nemathelminthes and Platyhelminthes, important for animal health care, and (2) ≥55% of sequence identity with other organisms. The gene ontology (GO) enrichment analysis (GOEA) was performed to study the level of gene regulation to IVMr and IVMs strains using Log Fold Change (LFC) filtering values ≥ 1 and ≥ 2. The upregulated-displayed genes obtained via GOEA were: 1993 (for LFC ≥ 1) and 1241 (for LFC ≥ 2) in IVMr and 1929 (for LFC ≥ 1) and 835 (for LFC ≥ 2) in IVMs. The enriched GO terms upregulated per category identified the intracellular structure, intracellular membrane-bounded organelle and integral component of the cell membrane as some principal cellular components. Meanwhile, efflux transmembrane transporter activity, ABC-type xenobiotic transporter activity and ATPase-coupled transmembrane transporter activity were associated with molecular function. Responses to nematicide activity, pharyngeal pumping and positive regulation of synaptic assembly were classified as biological processes that might be involved in events related to the anthelmintic resistance (AR) and nematode biology. The filtering analysis of both LFC values showed similar genes related to AR. This study deepens our knowledge about the mechanisms behind the processes of *H. contortus* in order to help in tool production and to facilitate the reduction of AR and promote the development of other control strategies, such as anthelmintic drug targets and vaccines.

## 1. Introduction

The blood-feeding nematode *Haemonchus contortus* is a highly prevalent and pathogenic parasite affecting small ruminants mainly in tropical and temperate grazing regions [1,2]. Haemonchosis is an economically important disease occasioning severe weight lost and stunted growth in small ruminants, resulting in immunosuppression and susceptibility to other diseases [3,4,5,6]. The clinical signs, anaemia and diarrhea, may indicate acute *H. contortus* infection that might cause death in small ruminants [6,7,8]. The nematode species *Cooperia* spp., *Ostertagia* spp. and *Haemonchus* spp. belong to the family Trichostrongylidae and have received special attention as biological models in ruminants because of the wide genetic diversity among nematode populations [9].

Anthelmintic resistance (AR) was notified in the three principal drug families. Different gene mechanisms have been associated with the anthelmintic resistance to the macrocyclic lactones (ML) family, specifically to ivermectin (IVM). One of these mechanisms has been associated with the regulation of the transcript level of some genes, for example, genes involved in xenobiotic transportation, i.e., Pgp 1, 2, 3, 4, 9, 10, 11, 12, 14 and 16, or genes associated with the metabolism of biotransformation enzymes (for instance cytochrome oxidase P-450, CYP14, glutathione S-transferases GST-4 and -10), and nucleotide changes attributed to ligand-gated chloride ionic channels, including GluClc^−^, Gly and GABA [10,11,12,13]. Likewise, proteomic, transcriptomic and genomic information through massive data analysis (RNASeq, DNASeq) and molecular tools (qPCR) in *H. contortus* have been important for understanding the adaptation mechanisms for survival of parasites associated with anthelmintic resistant, pathogenicity, immunogenicity, as well as for understanding the parasite–host interaction [14,15,16].

In this context, preliminary studies in Mexico have characterized two strains of *H. contortus*, one susceptible and the other resistant to ivermectin, designated as IVMs and IVMr, respectively. The analysis of these two strains using the P-gp relative gene expression through RT-qPCR lead us to identify their AR status.

Both strains were collected from naturally infected sheep and have been used as biological/gene references for IVM resistance studies [17]. Although other strategies of control remain currently under study, the transcriptome of gastrointestinal nematodes (GIN), i.e., *Haemonchus*, may contribute to the scientific knowledge about a number of genes with potential use to improve the use of recombinant vaccines. It can also contribute to our understanding of anthelmintic mechanisms and genes involved in nematode pathogenesis, among others [13,15,16]. Due to the importance of the genus *Haemonchus*, expanding our knowledge about the functional genes of this nematode is crucial for nematode control in grazing flocks and herds [16,18,19]. The objective of this study was therefore to assemble the transcriptome of *H. contortus* species as an important nematode model to contribute to the knowledge about the differential gene expression profile between two Mexican *H. contortus* strains under different AR statuses: one susceptible and the other resistant to ivermectin (IVMs and IVMr, respectively).

## 2. Materials and Methods

### 2.1. History of Haemonchus contortus Isolates

The two *H. contortus* strains, IVMs and IVMr, were phenotypically characterized according to their AR in sheep. The *H. contortus* IVMs strain was originally obtained from a naturally infected lamb at “Las Margaritas Ranch” in Hueytamalco village, Puebla State, Mexico, in 1990 [20]. Since then, this strain has been preserved under cryopreservation conditions without any IVM exposure. It has been refreshed many times by the Mexican government via passes in lambs for experimental purposes at the National Centre for Disciplinary Research in Animal Health and Innocuity from INIFAP-Agricultura. On the other hand, the IVMr strain was obtained from a naturally infected grazing sheep from a tropical region in the Salto de Agua municipality, Chiapas, Mexico [21]. Recently, the lethal concentration of IVM (as an analytical-grade commercial reagent) against infective larvae (L_3_) of both *H. contortus* strains was estimated. Data showed significant differences between both *H. contortus* L_3_ strains (*p* < 0.01), with a lethal effect of 79.22% for the IVMs strain at a concentration of 11.42 mM, whereas the IVMr strain was only slightly affected (5%) by the different assessed concentrations, which ranged from 1.43 and 11.42 mM [17].

#### Parasites

The *H. contortus* strains were obtained from sheep previously infected with larvae obtained from monocultures of the parasite; either IVM resistant or susceptible. Sheep feces containing eggs of the parasite were collected directly from the rectum of these animals, and monocultures were elaborated to obtain infective larvae. The experimental animals used in the present study were free of parasites and were infected with infective larvae from monocultures. In this way, we were completely sure that no other parasites were present in these animals. Likewise, our strains have been conserved in cryopreservation since 1990 [20] and 2020 [17]. One sheep was orally infected with 350 *H. contortus* L_3_ (IVMs) per kg BW, and the other sheep had received a similar infection with the same number of larvae; however, this animal was infected with the IVMr strain. Once the presence of the parasite eggs in the feces of the infected animals was identified, animals were slaughtered to obtain adult stages of the parasite from the abomasum of each animal infected with each strain. Animals were maintained under strict guidelines of animal welfare, following the criteria of the Official Mexican Standard Policies for caring and handling of experimental animals (NOM-051-ZOO-1995, NOM-033-Z00-1995 and NOM-062-ZOO-1999). The guidelines for experimental animal care were established by the Institutional Board of the National Autonomous University of Mexico, protocol number MC-2018/2-14. Female and male adult nematodes were collected and separated, and only males were used for the present study, following the procedure described by Reyes-Guerrero et al., 2020 [17]. Briefly, adult male parasites were collected from the sheep abomasum and decontaminated with sterilized PBS 0.15 M and 0.01 sodium azide and kept in 1.5 mL tubes containing 100 nematodes with an RNALater stabilization solution (Ambion™, Carlsbad, CA, USA) per tube (~20 aliquots) at −20 °C until they were used.

### 2.2. RNA-Seq

#### 2.2.1. Preparation of *Haemonchus contortus* Total RNA for Transcriptome Sequencing

**Figure 1:**
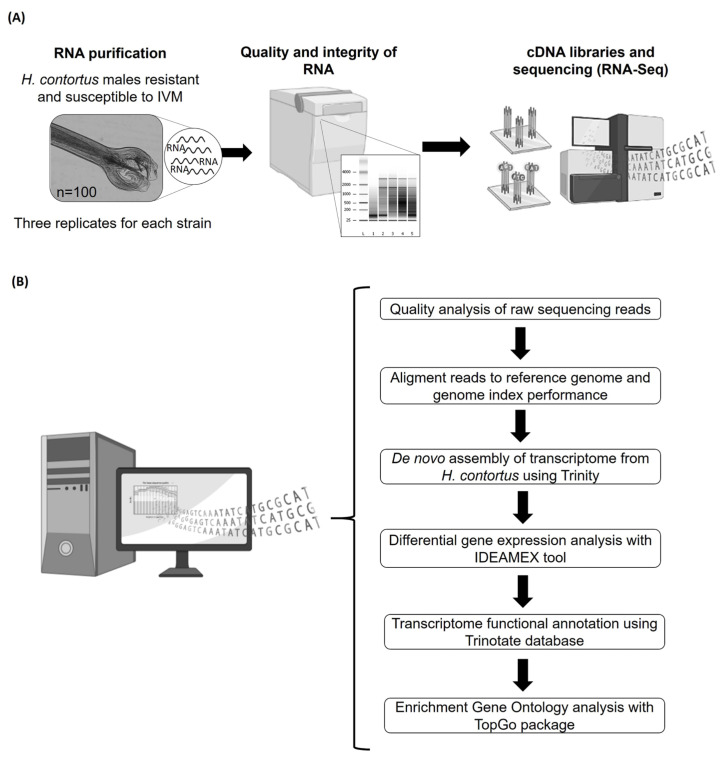
Summary and workflow diagram of experimental and bioinformatics to perform RNA-Seq analysis of two Mexican *H. contortus* strains resistant or susceptible to ivermectin. (**A**) Workflow of experimental and technical processes of RNA sequencing for *H. contortus strains*. (**B**) Bioinformatics analysis from de novo assembly of two *H. contortus* transcriptome strains (partial images supported by biorender.com).

The experimental design and the bioinformatic process are shown in Figure 1. Three 1.5 mL plastic tubes each containing 100 *H. contortus* adult males of each strain, either IVMs or IVMr, were used [22,23]. Extraction and purification of the total RNA were performed for each sample using a Trizol^®^ reagent (Thermo Fisher Scientific, Carlsbad, CA, USA). The disruption of adult nematodes was carried out using MagNa Lyser^®^ equipment (Roche, Mannheim, Baden-Württemberg, Germany). Total RNA was collected and purified with chloroform and isopropanol and then precipitated with 75% ethanol [17,24]. The RNA concentration was estimated at 3 µg using spectrophotometry (Nanophotometer, Implen N-80, Westlake Village, CA, USA). In addition, RNA purity and integrity were assessed via electrophoresis using 1% agarose gel, stained with ethidium bromide and determined via fluorometry using a Bioanalyzer 2100 according to the instructions of the manufacturer (Agilent, Santa Clara, CA, USA). This procedure was performed to achieve an RNA Integrity Number (RIN) ≥ 6 that we consider sufficient for our assay.

#### 2.2.2. Preparation of cDNA Libraries for the Sequencing Platform

The cDNA libraries were constructed from the extraction of total RNA per technical replicate for each *H. contortus* strain at the National Institute of Genomic Medicine (INMEGEN, Cd. México, México). The two following Illumina commercial prep kits were acquired: TruSeq RNA Library and TruSeq Stranded mRNA Library (Ilumina, San Diego, CA, USA). These kits were used to collect the RNA messenger (mRNA) molecules, for cDNA synthesis, for cDNA fragmentation and for the hybridization of sequence adapters. Once the cDNA libraries of each *H. contortus* strain were constructed, paired-end (PE) sequencing was performed in 76-bp inserts (2 × 76) on the Illumina NextSeq500 platform.

#### 2.2.3. Assembly, Annotation and Differential Expression Analysis

De novo assembly was generated using whole-transcriptome sequencing (RNA-Seq), and differential gene expression (DGE) analysis was performed using unigenes transcript reads between *H. contortus* IVMs and IVMr strains. Nucleotide sequence data reported in this paper are available in the GenBank™ database under the accession number BioProject ID: PRJNA877658. The study was performed in the computational cluster of high efficiency at the Unit of Massive Sequencing and Bioinformatic at the Biotechnology Institute, UNAM (UUSMB, IBT), following commands via Security Shell (SSH). The FastQC v. 0.11.8 program and the TrimGalore software were used to analyze the sequencing quality [25,26]. Throughout this process, repetitive and sequence adapters were trimmed with the TrimGalore and CutAdapt v. 1.16 software [26,27], respectively. The mRNA read sequences were aligned with the *H. contortus* nematode genomes of the references, MHco3 (ISE) and NZ_HCo_NP [16,22,28,29], using initially the Smalt v. 0.7.4 program [30]. However, the result for the alignment (56.11%) was considered as a low-mapped read between the Mexican *H. contortus* strains and the reference genomes. For this reason, the bowtie algorithm [31] was used to confirm the percentage, which showed a similar result. Due to this observation, it was decided to perform the assembly de novo of transcript sequences from *H. contortus* strains with the Trinity software v. 3.0 [32]. In this way we avoid losing 43.89% of transcripts information using 56.11% of the alignment obtained from the reference genomes. So, we think that this information could additionally contribute to the knowledge of the nematode transcriptome.

The level of *H. contortus* expression per strain was estimated using the number of transcript reads per million (TPM) aligned at each exon in the table counts and by using the RSEM software tool [33] (Data Supplementary S1). In addition, DGE analysis was carried out among strains via the DESeq2 algorithm [34], using a *p* value of ≤ 0.05 and log2 fold change (LFC) = 1. This algorithm was performed through the Integrated Differential Expression Analysis MultiEXperiment (IDEAMEX) web server [35]. The *H. contortus* IVMs was selected as a control for DGE analysis. Likewise, transcriptome functional annotation was performed using the intersection of differentially expressed transcripts’ (*p* ≤ 0.05) data from the DESeq2 algorithm and Trinotate repository. Trinotate was considered as an “ad hoc” comprehensive annotation sequence database for de novo-assembled transcriptome studies [36]. Thus, data were used for the enrichment analysis of gene ontology terms [37].

#### 2.2.4. Gene Ontology Enrichment Analysis (GOEA)

**Figure 2:**
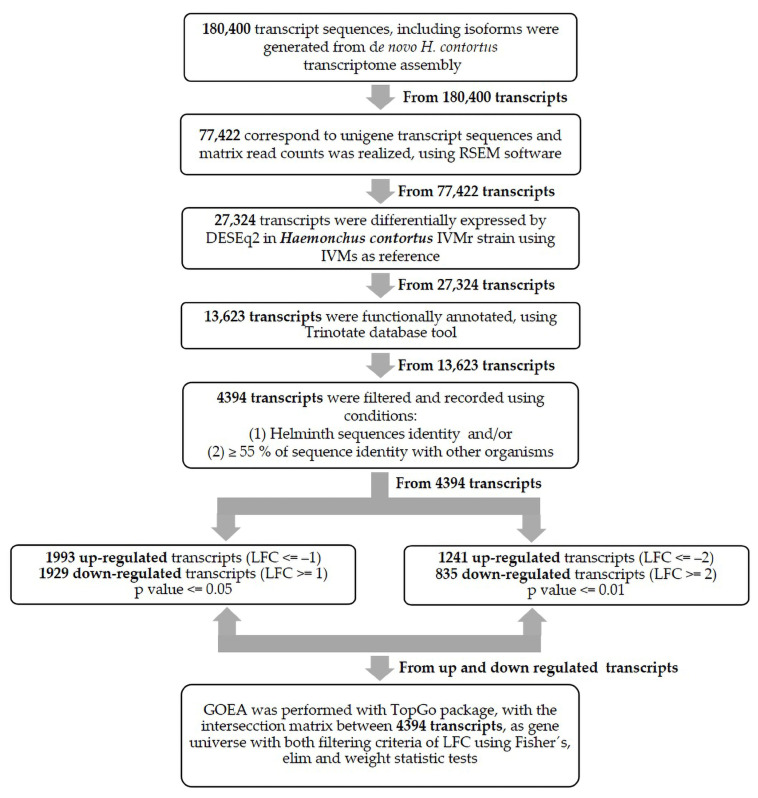
Workflow to perform differential expression analysis and gene ontology enrichment between *H. contortus* resistant and susceptible to ivermectin Mexican strains through DESeq2 and TopGo packages.

The GOEA was performed using 4394 transcript sequences obtained from the functional annotation of differentially expressed transcripts, corresponding to helminths (Nemathelminthes & Platyhelminthes phylum) sequences and/or ≥55% of sequence identity with other organisms. Then, the transcript reads, mapped from the GO terms, were analyzed through the Trinotate database. Trinotate uses of a number of different satisfactory reference methods for functional annotation, including an homology search for known sequence data (BLAST+/SwissProt/Uniref90), protein domain identification (HMMER/PFAM), protein signal peptide and transmembrane domain prediction (signalP/tmHMM) and a comparison to currently curated annotation databases (EMBL Uniprot eggNOG/GO Pathways databases). In addition, helminth data and their identity with other organisms’ intersections were filtered and recorded using two filtering criteria based on LFC values to perform the *H. contortus* gene ontology analysis: (a) log2 (fold) ≤ −1 for upIVMr/downIVMs and log2 (fold) ≥ 1 for downIVMr/upIVMs (*p*-value ≤ 0.05) and (b) log2 (fold) ≤ −2 for upIVMr/downIVMs and log2 (fold) ≥ 2 for downIVMr/upIVMs (*p*-value ≤ 0.01) Figure 2 shows the workflow to carry out DGE and GOEA studies. The TopGo package enrichment analysis used in these studies includes Fisher’s exact test, elim and weight statistical analysis [38].

## 3. Results and Discussion

### 3.1. Transcriptome Assembly and Features

*Haemonchus contortus* sequencing synthesis, displayed by the llumina platform, had 47,337,370 PE mean sequences per replicate for both IVMs and IVMr strains. The transcriptome-level assembly coverage was 150× using 76-pb sequence fragments. The final assembled *H. contortus* transcriptome (GenBank BioProject ID: PRJNA877658) size was 126,974,821 bp (~127 Mbp), integrated via 41,748,209 nucleotide sequences and displaying 180,400 sequences with an overall 44.4% GC content, including isoforms of which 77,422 transcript sequences correspond to unigenes (Table 1). From the total analyzed unigenes in our study (77,422), close to 22,066 transcripts constitute 28.5% of the annotated genes identified in database analyses. It is important to consider that 71.5% of genes still remain without annotation. So, more studies will be required regarding these transcript sequences in order for them to be included into the database network.

In addition, the 22,066 protein-coding genes recorded in this study showed a similar number of transcripts with other *H. contortus* strains such as MHco3 (ISE), NZ_Hco_NP (v1.0) and McMaster (HCON_v4), with 19,623, 22,341 and 23,610 coding genes, respectively, notified in the three genomes reported previously for this nematode [16,22,28,29]. To our knowledge, this is the first record about a transcriptome assembly of *H. contortus* Mexican strains differing in their anthelmintic susceptibility/resistance genomic level to IVM. Our results showed a high-quality assembly of *H. contortus* strains, allowing gene identification and comparison with other gene profiles associated with AR and other important biological functions.

### 3.2. Functional Transcription Annotation of H. contortus

Prior to the *H. contortus* transcriptome assembly, both strains were aligned with the *H. contortus* reference genomes; our results showed 56.11% mapped reads with respect to the genomes described by Doyle et al., 2020 (MHco3(ISE)), [16] and Palevich et al., 2019 (NZ_HCo_NP) [29]. Considering that our percentage mapped reads after aligning might be limited, we assumed that performing a de novo transcriptome analysis using *H. contortus* as one organism with different ivermectin resistance statuses, IVMs and IVMr, might provide more information about the biological processes of the nematode through gene functions. Additionally, the quality of the *H. contortus* massive sequences was compared with the one obtained from the *Ovis aries* genome (BioProject ID PRJNA739192) to confirm that transcript reads (=0.39%) corresponded to the nematode data but not to host data. On the other hand, our results about the transcript assembled displayed 77,422 total transcript sequences, with nucleotide fragment sizes ranging between 200 and 7700 bp (Table 1).

Likewise, from the 77,422 gene sequences, 60.2% showed size fragments lower than or equal to 500 bp; meanwhile, 39.8% of sequences are fragments with more than 500 bp. The analysis of transcript sequences provides information that could be related to important biological functions, such as reproduction, nutrition, xenobiotic detoxification and immune evasion in order to survive into the host [22]. It is important to mention that after obtaining data about genomes or transcriptome sequences, a meticulous analysis is crucial for accurately interpreting the generated information [16,29,39].

Because these strains belong to same genus/species, after we analyzed the number of total coding genes in our study, we obtained a 776-bp mean of the transcript size from both Mexican *H. contortus* strains; this transcript size was similar to the one reported by Pavelich et al., 2019, who obtained 666-bp transcriptional size described for a *H. contortus* strain from New Zealand [29]. Early studies suggested the use of the *H. contortus* transcriptomic profile to identify significant gene sequences with potential use as targets for novel vaccination agents [15,19,28]. The transcriptome profile could also contribute to searching for an AR reduction strategy, based on the genetic mechanisms of adaption associated with anthelmintic drugs pressure and the host–parasite interaction [40,41,42]. For example, 2825 and ~100,000 sequences in *H. contortus* adult males and in infective larvae, respectively, were identified via Serial Analysis of Gene Expression (SAGE) and EST analysis; these expressed transcript sequences were associated with different biological functions of the parasite [40,43].

### 3.3. Differential Gene Expression between Two Mexican H. contortus Strains

**Figure 3:**
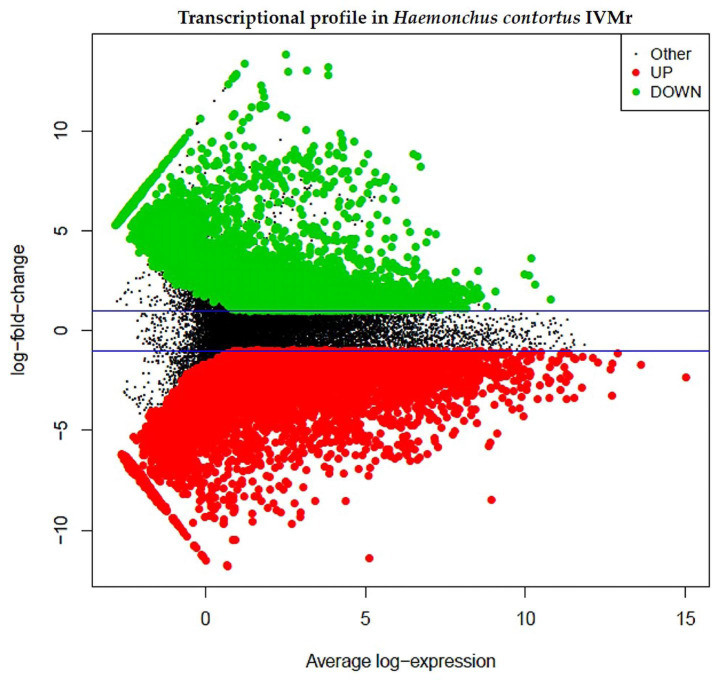
Transcriptional profile of differentially expressed genes from *H. contortus* resistant to ivermectin. Note: An IVM *H. contortus* susceptible strain was used as control and analyzed with the DESeq2 algorithm through the IDEAMEX web server.

From the counting matrix using 77,422 transcripts, 27,324 differentially expressed (DE) genes were obtained from two *H. contortus* strains. Figure 3 shows the transcriptional profile of DE genes in the IVMr strain with respect to the IVMs strain. Subsequently, the sequence data from the 27,324 transcripts DE were analyzed and functionally annotated, displaying 13,623 DE gene sequences from both *H. contortus* strains (Data Supplementary S2). In addition, the analysis of this information could be important to identify the mode of action of anthelmintic drugs from different families with similar activity against GIN, such as IVM and Levamisole (Imidazotiazol family). The toxicity of these drugs was observed on the nematode neuronal receptor and on the chloride ionic channel genes *(rAch* and *GluCl^−^*) and has been associated with AR [7,14]. Understanding the mechanisms of action of anthelmintic drugs as well as the mechanisms of parasite adaptation to their hosts can lead us to propose methods that maintain the efficacy of these drugs [6,7,9,14].

Thus, more studies comparing *H. contortus* IVMr and IVMs strains are necessary to provide specific gene profile information from the transcriptome related to AR. The knowledge about *H. contortus* genes supported by a genome is important to identify differential gene expression levels and mutations, specifically in *H. contortus* resistant to the IVM drug. Some of these mutations were identified in glutamate-gated chloride (GluCl^−^) channels such as avr-14 [44] and Hco-glc-3, 5, 37 genes [45,46,47]. In addition, an increase in the level of the relative expression was observed in the ABC transmembrane transporters family, such as with P-glycoprotein, for e.g., Hco-pgp-2. 3, 4, 9, 10 and 16 genes were reported in other studies using different *H. contortus* strains [17,45,48,49]. In addition, from the 13,624 DE genes recognized via a sequence homology search in the Trinotate database, 4394 gene sequences were found to belong to Nemathelminthes and Platyhelminthes phyla and other organisms with sequences that shared ≥55% identity with *H. contortus* strains from this study.

It is important to mention that from 4394 transcripts found, 72.25% showed homology with the free-living nematode *Caenorhabditis elegans* (=3175 sequences). In contrast, 9.85% corresponded to Strongylida suborder nematodes (=433 sequences), which includes parasitic nematodes affecting human, rodents and different domestic animals (*Ancylostoma*, *Nippostrongylus, Haemonchus* and *Ostertagia*). The remaining 17.9% of the gene sequences had homology with other helminths species, i.e., nematodes (*Ascaris* and *Anisakis*), trematodes (*Schistosoma*) and cestodes (*Echinococcus*). This information showed the importance of the transcriptome comparative studies of Mexican isolates of *H. contortus* with respect to other GIN populations because it could contribute to improve the efficacy of novel control methods such as vaccination and preserve the anthelmintic drug activity through the inhibition of genes associated with AR, such as in the competitive substrate studies [39,50,51].

Due to the high homology percentage between *H. contortus* and *C. elegans*, numerous studies have focused on studying drug resistance mechanisms, control strategies and the biology of different genera/species of the phylum Nematoda, including other free-living nematodes, as well as human, plant and animal parasites [51,52,53]. However, other genes may not be present in *C. elegans* or *H. contortus*, and perhaps these genes do not necessarily have an important biological impact on AR. Nevertheless, some genes found in *C. elegans* make this free-living nematode an important biological model. For example, genes such as *Hco-pgp-16* and *Cel-glc-1* have been identified in both *H. contortus* and *C. elegans* nematodes; however, they have not been found as orthologue genes in these two nematodes and might therefore be responsible of other specific biological functions [54,55].

### 3.4. Gene Ontology Enrichment Analysis (GOEA)

The GOEA analysis was performed with 4394 annotated and differentially expressed genes using both LFC filtering values. These genes were associated with the two criteria previously cited (which are helminths and/or which share ≥55% of sequence identity with other organisms) used to analyze the transcriptome of *H. contortus*. Table 2 included the comparative analysis between *H. contortus* IVMr/IVMs strains for each LFC filtering value. Due to the importance of AR in *H. contortus*, the upregulated genes from IVMr/IVMs were analyzed with their respective gene ontology terms (GO) and associated with the GOEA study. The analysis for each LFC value to *H. contortus* strains displayed differences between the categorization of GO terms in cellular components (CC), molecular functions (MF) and biological processes (BP) (Data Supplementary S3–S6). The analysis of LFC ≥ 1/≥ 2 showed 1993/1241 genes for the IVMr strain, respectively. Meanwhile, 1929/835 upregulated genes corresponded to the IVMs strain, for the same LFC values, respectively. These results showed a decreased number of unigenes using the LFC ≥ 2 value. Nevertheless, most of the upregulated genes associated with AR were conserved for both LFC filtering analysis values. These results are important key findings in relation to AR mechanisms. For instance, the group of ATPase-couple transmembrane transporter genes (GO:0042626) and the pharyngeal nematode pumping genes (GO: 0043050) were found with the LFC ≥ 1 filtering analysis, although these two groups of genes were not identified using the LFC ≥ 2 value. However, other relevant genes were identified with this LFC ≥ 2 value, such as the group of genes involved in the regulation of skeletal muscle contraction (GO:0014722) and locomotion (GO:0040017), which are part of the IVM mechanism to induce the muscle paralysis followed by the death of the nematode [12]. Interestingly, the group of the *unc* gene family (GO:0040017) was identified in the IVMr strain. Previous notifications about the *unc* gene family have been related to the Imidazotiazole anthelmintic family and also to ivermectin resistance in *H. contortus* [7,12,15]

Table 3 and Table 4 show some GO terms per category that were upregulated and related with metabolic and detoxification processes and associated with AR mechanisms in the IVMr strain for both LFC filtering values. For instance, MF included: efflux transmembrane transporter activity (GO:0015562), ABC-type xenobiotic transporter activity (GO:0008559) and ATPase-coupled transmembrane transporter activity (GO:0042626). Likewise, BP terms were associated with response to nematicide activity (GO:0093002), pharyngeal pumping (GO:0043050) and positive regulation of synaptic assembly (GO:0045887). In addition, some of them showed relations with host and parasite interactions due to immunology activity and sexual functions, among other factors. Such gene enrichment could be of importance for the study of ruminant nematodes as well as for other important parasites from the phylum Nematoda. For instance, Abubucker et al., 2009 [56], identified sequence transcripts related to the early stage nematodes that play an important role in the host–parasite interaction. Other nematodes, such as *C. elegans*, have been considered as important biological models in comparative studies, for example, *H. contortus* and *Ascaris suum* (a parasite of pigs) that are involved in neuromuscular physiology [50] and in the secretion of a novel class of endogenous non-coding RNAs called circular RNAs (circRNAs) [57].

Finally, the CC terms displayed the presence of intracellular structures and an intracellular membrane-bounded organelle (GO:0043231) and were also integral components of the cell membrane (GO:0016020, 0016021). These terms play important roles in the parasite development and in the metabolism of anthelmintic drugs [50,58].

On the other hand, the GO terms enriched and upregulated in *H. contortus* IVMs using the analysis of both LFC values showed conservation of genes with similar function in the three GO terms, most of them related to the development, structure and biology of this parasite (Table 5). For instance, nutrient reservoir activity (GO:0045735) and gap junction hemi-channel activity (GO:0055077) were some of the MF terms found in this study. Likewise, some of the terms for the BP were involved in the morphogenesis of the embryonic digestive tract and vulval development (GO:0048557; 0040025). The majority of CC terms were associated with mechanisms for cellular development, such as GO: 0005730, GO: 0022625, GO: 0005840 and GO:0000932, corresponding to the nucleolus, cytosolic large ribosomal subunit, ribosome and P-body, respectively. The latter refers to cytoplasm where mRNAs may be inactivated by decapping or perhaps other mechanisms [59,60]. These GO terms could be crucial to nematode survival, either at the free-living or parasitic stage.

The novel information presented in this study suggests the presence of a high genetic diversity in different *H. contortus* populations, and such diversity could be due to different factors, including anthelmintic drug pressure, nematode prevalence in their environments and geographical regions; it can also be the result of diverse factors associated with host–parasite interactions. The species *H. contortus* is one of the main nematodes that have developed genetic properties that enable them to tolerate anthelmintic drugs and to evade the host immune response through specific up/downregulation genes [16,50]. For instance, immune and detoxifying mechanisms related to parasitic nematodes are regulated by specific genes, such as the P-gp (Hco-pgp-3, 16) of *H. contortus*, which interacts with eosinophils related to the immune response, and these P-gps may increase the relative expression related to AR [61]. Interestingly, most of the MF genes identified in the present study correspond to the ABC-transporter family, which are involved in *H. contortus* IVMr, such as *ced7*, *wht-1* and *mrp2* [23], as well as the *nhr-8* gene, which has been reported as a regulator of detoxification genes in *H. contortus* and *C. elegans* pressured with the IVM drug [62]. Other important genes, such as those associated with the serine protease family (GO:0008236, GO:0004177), appear to be important as potential vaccine candidates, as has been notified from the nematode *Brugia malayi* (i.e., Bm-ALT and Bm-VAL-1) [63]. With these examples, a database of novel transcriptomes could expand our knowledge about nematode biology through the functional annotation of genes and could be directed to obtain future vaccines, molecular targets for competitive studies to inhibit AR receptors and molecular markers for AR diagnosis [23,50,58].

## 4. Conclusions

The present study describes, for first time, the de novo transcriptome assembled and obtained from two Mexican *H. contortus* strains with different ivermectin resistance/susceptible genotypic profiles. The results obtained from this study allow us to identify the relevant genetic differences between *H. contortus* strains, providing information from different groups of genes and allowing us to study and to improve anthelmintic efficacy via molecular strategies. These improvements could include gene receptor inhibition, RNA interference and establishing methods to identify molecular markers useful for AR diagnosis. Finally, this study potentially contributes the tools to design other control strategies, i.e., vaccines and new drug gene targets. The analysis of information about *Haemonchus* sp. transcriptome is crucial to understand the up/down gene regulation in nematode biology associated with AR and to other mechanisms involved in the host and parasite interaction. In addition, some interesting results, such as the transcriptome size (127 Mbp), could be explored to study GO terms such as the molecular function of unigenes of the ABC transporter family, ATPase-coupled transmembrane transporter activity and the unigene family that are involved with ivermectin and levamisole resistance to anthelmintic drugs. In addition, other sets of genes associated with biological functions should be studied based on their transcriptomic profile. For instance, cysteine-type peptidase family genes involved in the biology and development of parasitic nematodes, cestodes and trematodes in order to survive—ie. *Hemonchus*, *Teladorsagia* and *Fasciola* (trematode of ruminant)—could be used as potential candidates for vaccines or to improve those vaccines that are under study. The transcripts from *Haemonchus* offer plenty of novel sequences that require more studying to observe other opportunities against parasites.

## Figures and Tables

**Table 1:** Summary of de novo transcriptome assembled analysis representative of two *Haemonchus contortus* Mexican isolates with different ivermectin resistance statuses.

Features and Statistics	
Status	High-quality transcriptome
Isolation source	Male adult nematode (ovine abomasum)
Assembly method	Trinity/RNA-Seq de novo assembly
Transcriptome coverage	150×
Sequencing technology	Illumina
Assembled transcriptome size (bp)	126,974,821 (~127 Mb)
Total number of unigen transcripts	77,422
All transcripts contigs N50 (bp)	1149
DNA G + C (%) content	44.4
DNA A + T (%) content	55.6

**Table 2:** Number of enriched gene ontology terms and upregulated genes of *Haemonchus contortus* (IVMr/IVMs) based on filtering criteria of different LFC and *p*-values.

Total of Enriched Gene Ontology Terms	LFC ≤ −1 and ≥ 1 (*p* ≤ 0.05)		LFC ≤ −2 and ≥ 2 (*p* ≤ 0.01)	
	Total of Upregulated Genes			
	IVMr	IVMs	IVMr	IVMs
	1993	1929	1241	835
Molecular Function	34	33	29	22
Biological Process	66	31	50	28
Cellular Component	25	29	17	10

LFC = Log2 Fold Change; IVMr/IVMs = *H. contortus* resistant to ivermectin/susceptible to ivermectin.

**Table 3:** Enriched gene ontology terms per category and up regulated genes in a *Haemonchus contortus* ivermectin resistant strain (IVMr) using LFC ≥ 1.

GO Term	Gene	Trinity ID Transcript	Fold Change	*p* Value	Definition Based on Gene Ontology Consortium [59,60]
Molecular Function terms					
GO:0015562 Efflux transmembrane transporter activity	PGP1	DN7767	7.60	3.97 × 10^−16^	Enables the transfer of a specific substance or related group of substances from inside of the cell to outside of the cell across a membrane
	PGP3	DN40711	2.62	0.00035	
GO:0008559 ABC-type xenobiotic transporter activity	PGP1	DN7767	7.60	3.97 × 10^−16^	Catalysis of the reaction: ATP + H_2_O + xenobiotic (in) = ADP + phosphate + xenobiotic (out)
	PGP3	DN40711	2.62	0.00035	
GO:0042626 ATPase-coupled transmembrane transporter activity	PGP1	DN7767	7.60	3.97 × 10^−16^	Primary active transporter of a solute across a membrane, via the reaction: ATP + H_2_O = ADP + phosphate, to directly drive the transport of a substance across a membrane. The transport protein may be transiently phosphorylated (P-type transporters), or not (ABC-type transporters)
	CED7	DN133	2.07	1.84 × 10^−7^	
	WHT1	DN5402	3.22	2.38 × 10^−16^	
	ABCB8	DN8534	1.32	0.00021	
	MRP2	DN14918	1.81	0.00046	
	ABCA4	DN48845	2.09	0.00262	
	ABCD2	DN5820	2.96	1.85 × 10^−9^	
Biological Process terms					
GO:0093002 Response to nematicide activity	PGP1	DN7767	7.60	3.97 × 10^−16^	Processes that result in a change in state or activity of a cell or an organism (movement, secretion, enzyme production, gene expression, etc.) as a result of a nematicide stimulus
	SEK1	DN2949	2.03	3.24 × 10^−10^	
	KGB1	DN7108	2.33	1.39 × 10^−12^	
	PMK1	DN7240	2.03	1.62 × 10^−10^	
	NSY1	DN4471	1.36	0.00035	
GO:0043050 Pharyngeal nematode pumping	NAS5	DN1871	3.55	4.97 × 10^−27^	Contraction and relaxation movements of the pharyngeal muscle that mediate feeding in nematodes
	PXL1	DN10500	9.06	6.63 × 10^−12^	
	MYO4	DN4583	1.29	0.00962	
	ATPB	DN2532	1.62	4.50 × 10^−7^	
	CSK1	DN2039	1.35	3.02 × 10^−6^	
GO:0045887 Positive regulation of synaptic assembly	BLI4	DN5118	2.69	3.99 × 10^−45^	Processes that activate or increase the frequency, rate or extent of synaptic assembly at neuromuscular junction
	CBPE	DN6090	1.32	6.43 × 10^−7^	
	UN104	DN30477	1.74	0.00083	
	NEC2	DN8717	1.15	0.00152	
GO:0008152 metabolic process	NHR-8	DN3547	1.15	6.12 × 10^−7^	Chemical reactions and pathways by which living organisms transform chemical substances. Additionally, they include macromolecular processes (DNA repair and replication, protein synthesis and degradation)
	YMX8	DN1007	2.91	1.19 × 10^−7^	
Cellular Components terms					
GO:0043231 Intracellular membrane-bounded organelle	NHR-8	DN3547	1.15	6.12 × 10^−7^	Organized structure of distinctive morphology and function, bounded by a single or double lipid bilayer membrane and occurring within the cell
GO:0016020 Membrane	PGP1	DN10250	9.94	1.29 × 10^−15^	A lipid bilayer along with all the proteins and protein complexes embedded in it and attached to it
	WHT3	DN10777	2.42	2.76 × 10^−10^	

**Table 4:** Enriched gene ontology terms per category and upregulated genes in a *Haemonchus contortus* ivermectin resistant strain (IVMr) using LFC ≥ 2.

GO Term	Gene	Trinity ID Transcript	Fold Change	*p* Value	Definition Based on Gene Ontology Consortium [59,60]
Molecular Function terms					
GO:0003777 Microtubule motor activity	HUM6	DN10330	3.25	1.94 × 10^−6^	Motor activity that generates movement along a microtubule, driven by ATP hydrolysis
	KLC	DN11269	8.36	1.94 × 10^−9^	
	KI26L	DN12477	1.71	1.98 × 10^−5^	
	KLP3	DN15949	1.34	0.00012	
	MYO1	DN19625	3.36	7.09 × 10^−6^	
	MYO3	DN32812	2.88	2.34 × 10^−17^	
	MYO4	DN4583	1.29	0.00962	
Biological Process terms					
GO:0014722 Regulation of skeletal muscle contraction	UNC89	DN2825	2.28	5.90 × 10^−7^	Processes that modulate the frequency, rate or extent of skeletal muscle contraction by changing the calcium ion signals that trigger contraction
GO:0007018 Microtubule-based movement	HUM6	DN10330	3.25	1.94 × 10^−6^	Microtubule-based processes that result in the movement of organelles, other microtubules, or other cellular components by polymerization or depolymerization of microtubules
	KLC	DN11269	8.36	1.94 × 10^−9^	
	KI26L	DN12477	1.71	1.98 × 10^−5^	
	KLP3	DN15949	1.34	0.00012	
	MYO1	DN19625	3.36	7.09 × 10^−6^	
	MYO3	DN32812	2.88	2.34 × 10^−17^	
	MYO4	DN4583	1.29	0.00962	
GO:0040017 Positive regulation of locomotion	UNC89	DN2825	2.28	5.90 × 10^−7^	Any process that activates or increases the frequency, rate or extent of locomotion of a cell or organism
	UNC80	DN13535	1.59	0.00046	
	UNC52	DN11715	1.24	0.00893	
	LRP	DN12558	3.02	0.00744	
	EPI1	DN15848	1.55	0.00384	
	UNC6	DN19598	2.03	0.00016	
	UNC22	DN2116	1.68	1.41 × 10^−6^	
Cellular Components terms					
GO:0016021 Integral component of membrane	PGP1	DN10250	9.94	1.29 × 10^−15^	A lipid bilayer along with all the proteins and protein complexes embedded in it and attached to it
	NPL21	DN10287	1.61	0.00057	
	VEM1	DN1029	2.04	2.12 × 10^−12^	
	SKAT1	DN1033	1.59	3.53 × 10^−10^	
	NHX9	DN1038	1.71	4.57 × 10^−9^	
	PGAP2	DN10392	3.09	5.80 × 10^−22^	

**Table 5:** Some enriched gene ontology terms per category and upregulated genes in a *Haemonchus contortus* ivermectin susceptible strain (IVMs).

GO Term	Gene	Trinity ID Transcript	Fold Change	*p* Value	Definition Based on Gene Ontology Consortium [59,60]
Molecular Function terms					
GO:0045735 nutrient reservoir activity GO:0005319 lipid transporter activity	VIT1	DN59905	4.87	0.00024	GO:0045735 Functions in the storage of nutritious substrates. GO:0005319 Enables the directed movement of lipids into, out of or within a cell, or between cells
	VIT2	DN60842	12.87	1.52 × 10^−9^	
	VIT4	DN68591	13.68	2.60 × 10^−59^	
	VIT5	DN53584	5.27	0.00042	
	VIT6	DN61483	8.20	1.90 × 10^−9^	
GO:0004129 cytochrome-c oxidase activity	COX1	DN43209	3.59	2.49 × 10^−8^	Catalysis of the reaction: 4 ferrocytochrome c + O_2_ + 4 H+ = 4 ferricytochrome c + 2H_2_O
	COX2	DN37556	7.87	3.64 × 10^−157^	
	COX3	DN59338	2.15	7.95 × 10^−8^	
	COX5A	DN6162	1.17	7.68 × 10^−16^	
	COX6A	DN3177	1.06	9.47 × 10^−17^	
GO:0055077 gap junction hemi-channel activity	INX3	DN7528	3.90	0.00054	A wide pore channel activity that enables the transport of solutes across the membrane via a gap junction hemi-channel. Two gap junction hemi-channels coupled together form a complete gap junction.
	INX7	DN11374	2.16	2.65 × 10^−12^	
	INX10	DN3762	1.19	2.21 × 10^−5^	
	INX14	DN20	1.01	0.00226	
Biological Process terms					
GO:0048557 embryonic digestive tract morphogenesis	LIN53	DN1263	2.25	2.62 × 10^−14^	The process in which the anatomical structures of the digestive tract are generated and organized during embryonic development.
	LIN35	DN4658	2.32	6.71 × 10^−7^	
	MIG5	DN5547	2.30	1.18× 10^−9^	
GO:0040025 vulval development	SQV7	DN2801	2.09	6.36 × 10^−9^	The process whose specific outcome is the progression of the egg-laying organ of female and hermaphrodite nematodes over time, from its formation to the mature structure.
	UNC84	DN2062	1.52	6.31 × 10^−7^	
	GLD3	DN1099	1.05	0.00104	
GO:0006458 de novo protein folding	TCPA	DN16687	1.39	1.92 × 10^−10^	The process of assisting in the folding of a nascent peptide chain into its correct tertiary structure.
	TCPB	DN7949	1.66	1.45 × 10^−7^	
	TCPH	DN2283	1.28	6.37 × 10^−6^	
GO:0090727 positive regulation of brood size	CDK4	DN12924	2.28	3.38 × 10^−10^	Any process that increases brood size. Brood size is the number of progenies that survive embryogenesis and are cared for at one time.
	CED9	DN1092	2.81	1.47 × 10^−9^	
	CCND	DN1960	2.46	3.47 × 10^−8^	
Cellular Components terms					
GO:0000932 P-body	PATR1	DN2981	1.37	4.87 × 10^−8^	A focus on the cytoplasm where mRNAs may become inactivated by decapping or some other mechanism. Protein and RNA localized to these foci are involved in mRNA degradation, nonsense-mediated mRNA decay (NMD), translational repression and RNA-mediated gene silencing.
	CNOT7	DN6681	1.28	2.45 × 10^−6^	

## Data Availability

Data are contained within the article and supplementary material. Nucleotide sequence data reported in this paper are available in the GenBank™ database under the accession number BioProject ID: PRJNA877658.

## Supplementary Materials

The following supporting information can be downloaded at: https://www.mdpi.com/article/10.3390/pathogens12030499/s1, Data S1: Matrix_RSEM.gene.counts; Data S2: fold-change of *H. contortus* resistant vs. susceptible to ivermectin; Data S3: gene ontology upregulated terms in *H. contortus* ivermectin resistant (IVMr), using LFC ≥ 1; Data S4: gene ontology upregulated terms in *H. contortus* ivermectin susceptible (IVMs), using LFC ≥ 1; Data S5: gene ontology upregulated terms in *H. contortus* ivermectin resistant (IVMr), using LFC ≥ 2; Data S6: gene ontology upregulated terms in *H. contortus* ivermectin susceptible (IVMs), using LFC ≥ 2.
