# Supplementary material for: Assembly and Analysis of Haemonchus contortus Transcriptome as a Tool for the Knowledge of Ivermectin Resistance Mechanisms
Source: Pathogens. 2023 Mar 22;12(3):499. doi: 10.3390/pathogens12030499 (PMC10059914; doi:10.3390/pathogens12030499)
Supplement: Supplementary file 1 [file pathogens-12-00499-s001.zip › S3_Upregulated GO terms in IVMr LFC1.pdf]

UPREGULATED ENRICHED GENE ONTOLOGY TERMS PER CATEGORY IN *Haemonchus contortus* IVERMECTIN RESISTANT STRAIN (IVMr), USING LFC ≥ 1 (p value ≤ 0.05)

CELLULAR COMPONENTS

Number	GO:ID	Term	Annotated	Significant	Expected	Classic Fisher	Elim Fisher	Top Fisher	Parentchild Fisher
1	0005576	Extracellular region	479	324	202.57	< 1e-30	1.9e-29	5.8e-29	< 1e-30
2	0005764	Lysosome	141	104	59.63	1.1e-14	1.1e-14	1.4e-18	1.000
3	0016021	Component of membrane	823	429	348.05	5.7e-11	1.2e-09	2.6e-11	0.469
4	0005856	Cytoskeleton	268	141	113.34	0.00026	0.09513	9.2e-11	2.0e-23
5	0045121	Membrane raft	58	48	24.53	2.1e-10	2.1e-10	2.1e-10	1.000
6	0005615	Extracellular space	172	104	72.74	6.7e-07	6.7e-07	6.7e-10	5.0e-07
7	0031143	Pseudo-podium	24	24	10.15	9.6e-10	9.6e-10	3.0e-08	7.2e-07
8	0016324	Apical plasma membrane	65	48	27.49	1.9e-07	1.9e-07	2.7e-07	0.091
9	0005886	Plasma membrane	464	271	196.23	5.2e-14	5.7e-06	3.4e-06	1.1e-06
10	0031430	M band	26	22	11	1.1e-05	1.1e-05	1.1e-05	9.6e-06
11	0005759	Mitochondrial matrix	166	87	70.2	0.00456	0.00456	3.4e-05	3.4e-13
12	0031966	Mitochondrial membrane	190	58	80.35	0.99977	0.99977	0.00012	0.539
13	0031234	Extrinsic component cytoplasmic	25	20	10.57	0.00013	0.00013	0.00013	6.5e-06
14	0045252	Oxoglutarate dehydrogenase complex	19	16	8.04	0.00021	0.00021	0.00021	3.6e-07
15	0005863	Striated muscle myosin thick filament	13	12	5.5	0.00025	0.00025	0.00025	0.186
16	0005581	Collagen trimer	27	21	11.42	0.00018	0.00018	0.00034	1.2e-10
17	0005788	Endoplasmic reticulum lumen	25	19	10.57	0.00063	0.00063	0.00063	1.2e-06
18	0031672	A band	44	37	18.61	1.1e-08	0.00039	0.00079	9.8e-09
19	0045177	Apical part of cell	73	56	30.87	1.6e-09	0.00090	0.00217	1.3e-09
20	0043231	Intracellular membrane-bounded	1993	656	842.85	1.00000	1.00000	0.00225	1.000
21	0016020	Membrane	1276	636	539.63	6.3e-12	0.72366	0.00386	1.5e-12
22	0005771	Multivesicular body	7	7	2.96	0.00240	0.00240	0.00567	4.7e-05
23	0031941	filamentous actin	6	6	2.54	0.00569	0.00569	0.00569	7.0e-05
24	0030139	Endocytic vesicle	18	11	7.61	0.08442	0.08442	0.00569	0.112
25	0016323	Basolateral plasma membrane	30	20	12.69	0.00595	0.00595	0.00595	0.566

MOLECULAR FUNCTIONS

Number	GO:ID	Term	Annotated	Significant	Expected	Classic Fisher	Elim Fisher	Top Fisher	Parentchild Fisher
1	0008234	cysteine-type peptidase activity	249	227	115.85	< 1e-30	< 1e-30	< 1e-30	5.0e-05
2	0008239	dipeptidyl-peptidase activity	65	31.17	10	8.5e-20	8.5e-20	8.5e-20	0.0078
3	0008236	serine-type peptidase activity	90	83	41.87	9.9e-21	1.4e-17	7.8e-19	0.0133
4	0004177	aminopeptidase activity	69	62	32.1	2.2e-14	2.2e-14	3.8e-16	0.6202
5	0008237	metallopeptidase activity	168	134	78.16	1.4e-19	4.0e-13	2.1e-14	0.9732
6	0004190	aspartic-type endopeptidase activity	99	81	46.06	2.4e-13	2.4e-13	2.4e-13	0.0257
7	0004222	metalloendopeptidase activity	83	63	38.62	3.3e-08	3.3e-08	3.3e-08	0.6586
8	0015562	efflux transmembrane transporter activity	23	23	10.7	2.1e-08	2.1e-08	4.5e-08	1.4e-07
9	0008559	ABC-type xenobiotic transporter activity	22	22	10.24	4.5e-08	4.5e-08	4.5e-08	1.0000
10	0004494	methylmalonyl-CoA mutase activity	19	19	8.84	4.6e-07	4.6e-07	4.6e-07	0.0062
11	0031419	cobalamin binding	19	19	8.84	4.6e-07	4.6e-07	4.6e-07	0.0012
12	0051015	actin filament binding	55	43	25.59	1.3e-06	1.3e-06	1.3e-06	2.3e-10
13	0003777	microtubule motor activity	40	34	18.61	4.6e-07	4.6e-07	1.8e-05	1.5e-05
14	0008270	zinc ion binding	186	114	86.54	2.3e-05	2.3e-05	2.3e-05	0.1373
15	0004197	cysteine-type endopeptidase activity	41	31	19.08	0.00013	0.00013	7.1e-05	0.9432
16	0042302	structural constituent of cuticle	26	20	12.1	0.00153	0.00153	0.00012	1.2e-08
17	0004715	non-membrane spanning protein tyrosine	25	21	11.63	0.00012	0.00012	0.00012	0.0017
18	0004332	fructose-bisphosphate aldolase activity	11	11	5.12	0.00022	0.00022	0.00022	0.0833
19	0019841	retinol binding	11	11	5.12	0.00022	0.00022	0.00022	0.0767
20	0004252	serine-type endopeptidase activity	18	16	8.37	0.00023	0.00023	0.00023	0.2221
21	0004591	oxoglutarate dehydrogenase (succinyl-tra...	18	16	8.37	0.00023	0.00023	0.00023	0.0316

22	0030976	thiamine pyrophosphate binding	18	16	8.37	0.00023	0.00023	0.00023	4.8e-05
23	0004471	malate dehydrogenase (decarboxylating)	17	15	7.91	0.00044	0.00044	0.00044	1.9e-05
24	0008948	oxaloacetate decarboxylase activity	17	15	7.91	0.00044	0.00044	0.00044	0.0385
25	0004656	procollagen-proline 4-dioxygenase activity	10	10	4.65	0.00047	0.00047	0.00047	0.2308
26	0090736	MATH domain binding	10	10	4.65	0.00047	0.00047	0.00047	2.8e-05
27	0031418	L-ascorbic acid binding	13	12	6.05	0.00075	0.00075	0.00075	7.7e-05
28	0042626	ATPase-coupled transmembrane transporter	85	61	39.55	1.6e-06	0.00826	0.00380	3.8e-06
29	0004658	propionyl-CoA carboxylase activity	7	7	3.26	0.00469	0.00469	0.00469	0.0278
30	0005524	ATP binding	611	313	284.27	0.00593	0.00593	0.00593	3.1e-09
31	0008017	microtubule binding	49	32	22.8	0.00595	0.00595	0.00595	0.3600
32	0019902	phosphatase binding	38	19	17.68	0.39310	0.39310	0.00718	0.0314
33	0030246	carbohydrate binding	86	59	40.01	2.4e-05	0.00132	0.00852	2.0e-07
34	0008013	beta-catenin binding	15	12	6.98	0.00866	0.00866	0.00866	0.0023

BIOLOGICAL PROCESSES

Number	GO:ID	Term	Annotated	Significant	Expected	Classic Fisher	Elim Fisher	Top Fisher	Parentchild Fisher
1	0006508	proteolysis	351	215	134.89	3.30E-20	3.40E-19	< 1e-30	1.40E-25
2	0045087	innate immune response	149	126	57.26	< 1e-30	< 1e-30	< 1e-30	0.00035
3	0030163	protein catabolic process	232	135	89.16	1.90E-10	1.90E-10	1.00E-17	3.80E-10
4	0070265	necrotic cell death	89	65	34.2	1.90E-11	1.90E-11	3.70E-14	6.20E-06
5	0006096	glycolytic process	43	21.9	29	1.10E-08	1.10E-08	8.60E-09	0.15108
6	0093002	response to nematicide	23	22	8.84	9.50E-09	9.50E-09	9.50E-09	0.08895
7	0008360	regulation of cell shape	35	30	13.45	9.20E-09	9.20E-09	2.10E-08	3.40E-06
8	0010038	response to metal ion	46	36	17.68	3.40E-08	5.90E-06	1.30E-07	0.00055
9	0038083	peptidyl-tyrosine autophosphorylation	23	21	8.84	1.70E-07	1.70E-07	1.70E-07	7.70E-06
10	0060298	positive regulation of sarcomere organiz...	19	18	7.3	3.70E-07	3.70E-07	3.70E-07	0.00016
11	0050829	defense response to Gram-negative bacter...	45	34	17.29	3.80E-07	3.80E-07	4.60E-07	0.26736
12	1904747	positive regulation of apoptotic process...	24	21	9.22	8.90E-07	8.90E-07	8.90E-07	0.00071
13	0045989	positive regulation of striated muscle c...	15	15	5.76	5.60E-07	5.60E-07	1.50E-06	0.00478
14	0018105	peptidyl-serine phosphorylation	54	38	20.75	1.60E-06	1.60E-06	1.60E-06	0.00106
15	0016477	cell migration	143	76	54.95	0.00018	0.02038	2.10E-06	0.98552
16	0007018	microtubule-based movement	69	47	26.52	4.30E-07	0.00512	2.20E-06	6.60E-05
17	0008152	metabolic process	2159	783	829.69	0.99992	1	4.50E-06	0.99992
18	2000057	negative regulation of Wnt signaling pat...	12	12	4.61	1.00E-05	1.00E-05	1.00E-05	2.80E-05
19	0015917	aminophospholipid transport	21	18	8.07	1.10E-05	1.10E-05	1.10E-05	3.20E-08
20	0007155	cell adhesion	92	60	35.36	1.20E-07	5.70E-06	1.80E-05	7.90E-10
21	0035023	regulation of Rho protein signal transdu...	11	11	4.23	111	2.60E-05	2.60E-05	0.00244
22	1901076	positive regulation of engulfment of apo...	27	21	10.38	119	3.40E-05	3.40E-05	0.05196
23	0007169	transmembrane receptor protein tyrosine ...	49	32	18.83	0.00011	0.00011	3.60E-05	0.04831
24	0006520	cellular amino acid metabolic process	138	68	53.03	0.00519	0.42765	6.30E-05	0.00024
25	0014722	regulation of skeletal muscle contractio...	10	10	3.84	6.90E-05	6.90E-05	6.90E-05	0.00137
26	1905552	positive regulation of protein localizat...	10	10	3.84	6.90E-05	6.90E-05	6.90E-05	2.20E-05

27	0045887	positive regulation of synaptic assembly...	16	14	6.15	7.30E-05	7.30E-05	7.30E-05	0.02505
28	1905905	pharyngeal gland morphogenesis	16	14	6.15	7.30E-05	7.30E-05	7.30E-05	1
29	0030317	flagellated sperm motility	13	12	5	8.40E-05	8.40E-05	8.40E-05	0.14286
30	1903356	positive regulation of distal tip cell m...	30	22	11.53	0.0001	0.0001	0.0001	0.05352
31	0040002	collagen and cuticulin-based cuticle dev...	33	26	12.68	2.40E-06	0.00011	0.00011	0.4249
32	0030241	skeletal muscle myosin thick filament as...	15	13	5.76	0.00017	0.00017	0.00017	0.17828
33	0040017	positive regulation of locomotion	84	54	32.28	1.00E-06	0.00118	0.00017	3.50E-06
34	0010172	embryonic body morphogenesis	49	32	18.83	0.00011	0.00011	0.00019	0.00021
35	0051603	proteolysis involved in cellular protein...	123	46	47.27	0.62863	0.62863	0.00024	1
36	0018996	molting cycle, collagen and cuticulin-ba...	47	34	18.06	2.10E-06	0.00011	0.00031	0.03443
37	0030048	actin filament-based movement	13	12	5	8.40E-05	8.40E-05	0.00046	0.01034
38	0019556	histidine catabolic process to glutamate...	8	8	3.07	0.00047	0.00047	0.00047	0.02935
39	0019557	histidine catabolic process to glutamate...	8	8	3.07	0.00047	0.00047	0.00047	0.02935
40	0050790	regulation of catalytic activity	96	34	36.89	0.76393	0.76393	0.00053	0.61813
41	0051017	actin filament bundle assembly	16	13	6.15	0.00058	0.00058	0.00058	9.70E-05
42	0048814	regulation of dendrite morphogenesis	12	11	4.61	0.0002	0.0002	0.00117	0.03152
43	0051296	establishment of meiotic spindle orienta...	10	9	3.84	0.00118	0.00118	0.00118	0.00988
44	0007060	male meiosis chromosome segregation	9	9	3.46	0.00018	0.00018	0.00122	1.70E-05
45	0019511	peptidyl-proline hydroxylation	10	9	3.84	0.00118	0.00118	0.00122	0.0035
46	0007605	sensory perception of sound	7	7	2.69	0.00122	0.00122	0.00122	0.05128
47	0031272	regulation of pseudopodium assembly	7	7	2.69	0.00122	0.00122	0.00122	0.03497
48	0045793	positive regulation of cell size	7	7	2.69	0.00122	0.00122	0.00122	0.07148
49	0048692	negative regulation of axon extension in...	7	7	2.69	0.00122	0.00122	0.00122	0.03497
50	0060279	positive regulation of ovulation	14	11	5.38	0.00263	0.00263	0.00263	0.08143

51	0040018	positive regulation of multicellular org...	39	24	14.99	0.00275	0.00275	0.00275	0.29822
52	1902075	cellular response to salt	9	8	3.46	0.00278	0.00278	0.00278	0.01567
53	1902473	regulation of protein localization to sy...	9	8	3.46	0.00278	0.00278	0.00278	0.01526
54	0051046	regulation of secretion	33	18	12.68	0.04311	0.04311	0.00317	0.22185
55	0006589	octopamine biosynthetic process	6	6	2.31	0.0032	0.0032	0.0032	0.11068
56	0009258	10-formyltetrahydrofolate catabolic proc...	6	6	2.31	0.0032	0.0032	0.0032	0.34286
57	0045604	regulation of epidermal cell differentia...	16	12	6.15	0.00324	0.00324	0.00324	0.18163
58	0043050	pharyngeal pumping	24	16	9.22	0.00461	0.00461	0.00542	1
59	0042338	cuticle development involved in collagen...	15	11	5.76	0.00646	0.00646	0.00646	0.7587
60	1990048	anterograde neuronal dense core vesicle ...	9	8	3.46	0.00278	0.00278	0.00649	0.0014
61	0072382	minus-end-directed vesicle transport alo...	8	7	3.07	0.00651	0.00651	0.00651	0.00886
62	0098609	cell-cell adhesion	34	21	13.07	0.00473	0.00473	0.00816	0.77673
63	0050830	defense response to Gram-positive bacter...	27	17	10.38	0.00826	0.00826	0.00826	0.94797
64	0055088	lipid homeostasis	8	7	3.07	0.00651	0.00651	0.00831	0.02912
65	0019433	triglyceride catabolic process	5	5	1.92	0.00834	0.00834	0.00834	0.15909
66	0030070	insulin processing	5	5	1.92	0.00834	0.00834	0.00834	1
