# Supplementary material for: Assembly and Analysis of Haemonchus contortus Transcriptome as a Tool for the Knowledge of Ivermectin Resistance Mechanisms
Source: Pathogens. 2023 Mar 22;12(3):499. doi: 10.3390/pathogens12030499 (PMC10059914; doi:10.3390/pathogens12030499)
Supplement: Supplementary file 1 [file pathogens-12-00499-s001.zip › S4_Upregulated GO terms in IVMs LFC1.pdf]

UPREGULATED ENRICHED GENE ONTOLOGY TERMS PER CATEGORY IN *Haemonchus contortus* IVERMECTIN SUSCEPTIBLE STRAIN (IVMs), USING LFC ≥ 1 (p value ≤ 0.05)

CELLULAR COMPONENTS

Number	GO:ID	Term	Annotated	Significant	Expected	Classic Fisher	Elim Fisher	Topgo Fisher	Parentchild Fisher
1	GO:0005730	nucleolus	126	106	57.97	2.1e-19	2.1e-19	1.0e-14	1.8e-09
2	GO:0005634	nucleus	1022	668	470.17	< 1e-30	1.9e-13	4.5e-14	3.9e-29
3	GO:0022625	cytosolic large ribosomal subunit	47	45	21.62	1.6e-13	1.6e-13	1.6e-13	0.46841
4	GO:0005840	ribosome	135	127	62.11	< 1e-30	9.8e-10	2.1e-09	8.4e-23
5	GO:0022627	cytosolic small ribosomal subunit	34	32	15.64	2.4e-09	2.4e-09	2.4e-09	0.63347
6	GO:0071011	precatalytic spliceosome	22	22	10.12	3.5e-08	3.5e-08	3.5e-08	0.01909
7	GO:0071013	catalytic step 2 spliceosome	43	40	19.78	5.3e-11	5.8e-08	5.8e-08	6.0e-07
8	GO:0005694	chromosome	145	105	66.71	4.3e-11	7.1e-06	1.1e-06	0.00038
9	GO:0032040	small-subunit processome	16	16	7.36	3.9e-06	3.9e-06	3.9e-06	0.32859
10	GO:0005654	nucleoplasm	187	141	86.03	4.3e-17	3.6e-06	9.9e-06	5.6e-17
11	GO:0000932	P-body	21	19	9.66	2.5e-05	2.5e-05	2.5e-05	0.01623
12	GO:0005686	U2 snRNP	12	12	5.52	8.8e-05	8.8e-05	8.8e-05	0.33498
13	GO:0000974	Prp19 complex	11	11	5.06	0.00019	0.00019	0.00019	0.01093
14	GO:0005681	spliceosomal complex	90	77	41.4	3.3e-15	0.00020	0.00020	0.61065
15	GO:0005829	cytosol	349	232	160.56	4.2e-16	6.0e-05	0.00039	6.0e-16
16	GO:0046540	U4/U6 x U5 tri-snRNP complex	10	10	4.6	0.00042	0.00042	0.00042	1.00000
17	GO:0071007	U2-type catalytic step 2 spliceosome	10	10	4.6	0.00042	0.00042	0.00042	0.31902
18	GO:0005682	U5 snRNP	9	9	4.14	0.00091	0.00091	0.00091	0.46798
19	GO:0016607	nuclear speck	29	22	13.34	0.00101	0.00101	0.00101	0.85240
20	GO:0016592	mediator complex	18	15	8.28	0.00129	0.00129	0.00129	0.61970
21	GO:0005743	mitochondrial inner membrane	150	90	69.01	0.00030	0.00247	0.00212	0.01023
22	GO:0030687	preribosome, large subunit precursor	15	13	6.9	0.00140	0.00140	0.00266	1.00000
23	GO:0005763	mitochondrial small ribosomal subunit	14	12	6.44	0.00267	0.00267	0.00267	0.11359
24	GO:0005665	RNA polymerase II, core complex	11	10	5.06	0.00268	0.00268	0.00268	0.13310
25	GO:0005832	chaperonin-containing T-complex	11	10	5.06	0.00268	0.00268	0.00268	0.07018
26	GO:0042555	MCM complex	7	7	3.22	0.00433	0.00433	0.00433	0.05688
27	GO:0005739	mitochondrion	479	240	220.36	0.02975	0.89645	0.00530	0.77504

28	GO:0005751	mitochondrial respiratory chain complex ...	10	9	4.6	0.00536	0.00536	0.00536	0.03119
29	GO:0019773	proteasome core complex, alpha-subunit c...	10	9	4.6	0.00536	0.00536	0.00536	0.10091

MOLECULAR FUNCTIONS

Number	GO:ID	Term	Annotated	Significant	Expected	Classic fisher	Elim fisher	Topgo fisher	Parentchild fisher
1	GO:0003735	structural constituent of ribosome	118	112	50.41	< 1e-30	< 1e-30	< 1e-30	4.9e-22
2	GO:0003723	RNA binding	362	287	154.66	< 1e-30	< 1e-30	1.7e-28	0.00039
3	GO:0003677	DNA binding	218	151	93.14	2.9e-16	1.1e-13	2.9e-16	0.98507
4	GO:0045735	nutrient reservoir activity	19	19	8.12	9.0e-08	9.0e-08	9.0e-08	9.0e-08
5	GO:0046982	protein heterodimerization activity	16	15	6.84	2.7e-05	2.7e-05	2.7e-05	0.00021
6	GO:0019843	rRNA binding	31	28	13.24	3.7e-08	3.7e-08	5.7e-05	0.08109
7	GO:0003746	translation elongation factor activity	22	18	9.4	0.00020	0.00020	0.00020	0.04135
8	GO:0003697	single-stranded DNA binding	17	15	7.26	0.00014	0.00014	0.00028	0.06108
9	GO:0044183	protein folding chaperone	13	12	5.55	0.00028	0.00028	0.00028	0.00028
10	GO:0005319	lipid transporter activity	26	20	11.11	0.00040	0.00040	0.00029	0.00010
11	GO:0003729	mRNA binding	50	39	21.36	3.3e-07	5.7e-05	0.00033	0.67367
12	GO:0003755	peptidyl-prolyl cis-trans isomerase acti...	18	15	7.69	0.00049	0.00049	0.00049	1.3e-07
13	GO:0019901	protein kinase binding	58	35	24.78	0.00485	0.00485	0.00079	0.33441
14	GO:0000993	RNA polymerase II complex binding	8	8	3.42	0.00110	0.00110	0.00110	1.00000
15	GO:0004360	glutamine-fructose-6-phosphate transamin...	8	8	3.42	0.00110	0.00110	0.00110	1.00000
16	GO:0004748	ribonucleoside-diphosphate reductase act...	8	8	3.42	0.00110	0.00110	0.00110	1.00000
17	GO:0003899	DNA-directed 5'-3' RNA polymerase activi...	17	15	7.26	0.00014	0.00014	0.00120	1.00000
18	GO:0004298	threonine-type endopeptidase activity	14	12	5.98	0.00121	0.00121	0.00121	9.2e-08

19	GO:0051082	unfolded protein binding	40	27	17.09	0.00129	0.00129	0.00129	0.00142
20	GO:0004129	cytochrome-c oxidase activity	35	24	14.95	0.00172	0.00172	0.00172	0.00907
21	GO:0004386	helicase activity	30	27	12.82	7.9e-08	0.00023	0.00181	2.3e-07
22	GO:0003730	mRNA 3'-UTR binding	16	13	6.84	0.00192	0.00192	0.00192	0.50452
23	GO:0042393	histone binding	22	18	9.4	0.00020	0.00020	0.00242	0.00022
24	GO:0003678	DNA helicase activity	11	11	4.7	8.5e-05	8.5e-05	0.00255	0.17876
25	GO:0008134	transcription factor binding	43	28	18.37	0.00242	0.00242	0.00280	0.00267
26	GO:0004693	cyclin-dependent protein serine/threonin...	15	12	6.41	0.00370	0.00370	0.00370	0.00015
27	GO:0008168	methyltransferase activity	47	39	20.08	1.4e-08	0.00380	0.00568	0.00073
28	GO:0005085	guanyl-nucleotide exchange factor activi...	6	6	2.56	0.00605	0.00605	0.00605	0.07933
29	GO:0017056	structural constituent of nuclear pore	6	6	2.56	0.00605	0.00605	0.00605	0.09753
30	GO:0055077	gap junction hemi-channel activity	6	6	2.56	0.00605	0.00605	0.00605	1.00000
31	GO:0070403	NAD+ binding	6	6	2.56	0.00605	0.00605	0.00605	0.00298
32	GO:0004713	protein tyrosine kinase activity	34	10	14.53	0.96235	0.96235	0.00624	0.60716
33	GO:0003676	nucleic acid binding	605	449	258.48	< 1e-30	0.00484	0.00777	< 1e-30

BIOLOGICAL PROCESSES

Number	GO:ID	Term	Annotated	Significant	Expected	Classic Fisher	Elim Fisher	Topgo Fisher	Parentchild Fisher
1	GO:0006412	translation	211	171	104.65	1.4e-22	1.1e-16	2.0e-22	5.4e-10
2	GO:0006260	DNA replication	62	58	30.75	5.2e-14	1.6e-08	3.1e-08	8.2e-06
3	GO:0006281	DNA repair	80	68	39.68	2.3e-11	9.5e-08	2.0e-06	0.00021
4	GO:0000398	mRNA splicing, via spliceosome	106	84	52.57	1.6e-10	1.1e-08	4.8e-06	0.68825
5	GO:0006364	rRNA processing	77	72	38.19	3.9e-17	1.4e-05	5.5e-06	0.05906
6	GO:0000027	ribosomal large subunit assembly	19	18	9.42	3.2e-05	3.2e-05	3.2e-05	0.28054
7	GO:0000462	maturation of SSU-rRNA from tricistronic...	17	17	8.43	6.4e-06	6.4e-06	5.2e-05	1.00000
8	GO:0006355	regulation of transcription, DNA-templat...	253	154	125.48	0.00011	0.00059	5.6e-05	0.79091
9	GO:0000470	maturation of LSU-rRNA	19	17	9.42	0.00031	0.00031	0.00021	0.91717
10	GO:0006048	UDP-N-acetylglucosamine biosynthetic pro...	12	12	5.95	0.00022	0.00022	0.00022	1.00000
11	GO:0048557	embryonic digestive tract morphogenesis	17	16	8.43	0.00012	0.00012	0.00022	1.9e-05
12	GO:0002181	cytoplasmic translation	36	30	17.86	2.6e-05	2.6e-05	0.00036	0.45258
13	GO:0006487	protein N-linked glycosylation	31	23	15.38	0.00452	0.00452	0.00056	0.00345
14	GO:0000028	ribosomal small subunit assembly	10	10	4.96	0.00089	0.00089	0.00089	0.32228
15	GO:0040025	vulval development	64	45	31.74	0.00056	0.00056	0.00147	0.00129
16	GO:0045892	negative regulation of transcription, DN...	65	39	32.24	0.05795	0.05795	0.00184	0.73258
17	GO:0006397	mRNA processing	154	123	76.38	2.0e-15	2.6e-06	0.00268	0.82291
18	GO:0006458	'de novo' protein folding	14	13	6.94	0.00081	0.00081	0.00286	0.01335
19	GO:0000381	regulation of alternative mRNA splicing,...	12	11	5.95	0.00288	0.00288	0.00288	0.15385
20	GO:0061077	chaperone-mediated protein folding	17	15	8.43	0.00103	0.00103	0.00328	0.02011
21	GO:0042273	ribosomal large subunit biogenesis	43	40	21.33	9.0e-10	0.00336	0.00336	0.31721
22	GO:0009263	deoxyribonucleotide biosynthetic process	12	12	5.95	0.00022	0.00022	0.00359	0.00866
23	GO:0000460	maturation of 5.8S rRNA	12	11	5.95	0.00288	0.00288	0.00361	0.82926
24	GO:0000413	protein peptidyl-prolyl isomerization	8	8	3.97	0.00363	0.00363	0.00363	2.3e-05
25	GO:0006189	'de novo' IMP biosynthetic process	8	8	3.97	0.00363	0.00363	0.00363	0.02222
26	GO:0006283	transcription-coupled nucleotide-excisio...	8	8	3.97	0.00363	0.00363	0.00363	1.00000
27	GO:0090727	positive regulation of brood size	8	8	3.97	0.00363	0.00363	0.00363	0.33333
28	GO:0006270	DNA replication initiation	9	9	4.46	0.00179	0.00179	0.00730	0.12399
29	GO:0006357	regulation of transcription by RNA polym...	157	84	77.87	0.17813	0.17813	0.00836	0.99981
30	GO:0006541	glutamine metabolic process	20	16	9.92	0.00522	0.00522	0.00855	0.00013
31	GO:0010501	RNA secondary structure unwinding	19	15	9.42	0.00858	0.00858	0.00858	0.33342
