# Supplementary material for: Assembly and Analysis of Haemonchus contortus Transcriptome as a Tool for the Knowledge of Ivermectin Resistance Mechanisms
Source: Pathogens. 2023 Mar 22;12(3):499. doi: 10.3390/pathogens12030499 (PMC10059914; doi:10.3390/pathogens12030499)
Supplement: Supplementary file 1 [file pathogens-12-00499-s001.zip › S5_Upregulated GO terms in IVMr LFC2.pdf]

UPREGULATED ENRICHED GENE ONTOLOGY TERMS PER CATEGORY IN *Haemonchus contortus* IVERMECTIN RESISTANT STRAIN (IVMr), USING LFC ≥ 2 (p value ≤ 0.01)

CELLULAR COMPONENTS

Number	GO:ID	Term	Annotated	Significant	Expected	Classic Fisher	Elim Fisher	Topgo Fisher	Parentchild Fisher
1	GO:0005764	lysosome	141	83	37.02	7.2e-17	7.2e-17	1.7e-20	1.00000
2	GO:0045121	membrane raft	58	40	15.23	8.3e-12	8.3e-12	8.3e-12	1.00000
3	GO:0005576	extracellular region	479	196	125.75	3.2e-14	6.8e-12	1.9e-11	1.9e-14
4	GO:0031143	pseudopodium	24	21	6.3	4.7e-10	4.7e-10	3.2e-09	4.8e-08
5	GO:0016021	integral component of membrane	823	270	216.05	9.9e-07	9.9e-07	1.2e-07	0.74798
6	GO:0016324	apical plasma membrane	65	35	17.06	1.7e-06	1.7e-06	1.4e-06	0.08379
7	GO:0005856	cytoskeleton	268	77	70.36	0.18694	0.45652	3.0e-06	2.1e-08
8	GO:0005759	mitochondrial matrix	166	65	43.58	0.00013	0.00013	3.1e-06	1.3e-12
9	GO:0005615	extracellular space	172	65	45.15	0.00044	0.00044	4.5e-05	0.00039
10	GO:0045252	oxoglutarate dehydrogenase complex	19	13	4.99	0.00014	0.00014	0.00014	2.0e-06
11	GO:0031430	M band	26	16	6.83	0.00015	0.00015	0.00015	0.00014
12	GO:0005771	multivesicular body	7	7	1.84	8.5e-05	8.5e-05	0.00032	5.9e-06
13	GO:0031941	filamentous actin	6	6	1.58	0.00032	0.00032	0.00032	1.9e-06
14	GO:0030139	endocytic vesicle	18	9	4.73	0.02614	0.02614	0.00032	0.05482
15	GO:0031966	mitochondrial membrane	190	36	49.88	0.99392	0.99392	0.00061	0.48087
16	GO:0031234	extrinsic component of cytoplasmic side	25	14	6.56	0.00148	0.00148	0.00148	0.00025
17	GO:0005581	collagen trimer	27	14	7.09	0.00387	0.00387	0.00387	1.3e-07

MOLECULAR FUNCTIONS

Number	GO:ID	Term	Annotated	Significant	Expected	Classic Fisher	Elim Fisher	Topgo Fisher	Parentchild Fisher
1	GO:0008234	cysteine-type peptidase activity	249	174	76.22	< 1e-30	< 1e-30	< 1e-30	0.04205
2	GO:0008239	dipeptidyl-peptidase activity	67	60	20.51	3.5e-24	3.5e-24	3.5e-24	0.00017
3	GO:0008236	serine-type peptidase activity	90	72	27.55	1.5e-22	6.8e-22	1.6e-23	0.00105
4	GO:0004190	aspartic-type endopeptidase activity	99	66	30.3	7.1e-14	7.1e-14	7.1e-14	0.02767
5	GO:0004177	aminopeptidase activity	69	49	21.12	3.6e-12	3.6e-12	3.6e-12	0.89963
6	GO:0008237	metallopeptidase activity	168	99	51.42	6.5e-15	4.3e-10	4.3e-11	0.98700
7	GO:0008559	ABC-type xenobiotic transporter activity	22	19	6.73	8.5e-08	8.5e-08	8.5e-08	0.42174
8	GO:0015562	efflux transmembrane transporter activit...	23	19	7.04	3.5e-07	3.5e-07	3.5e-07	2.0e-07
9	GO:0004197	cysteine-type endopeptidase activity	41	28	12.55	6.5e-07	6.5e-07	6.8e-07	0.33151
10	GO:0004222	metalloendopeptidase activity	83	46	25.41	1.8e-06	1.8e-06	1.8e-06	0.84860
11	GO:0004471	malate dehydrogenase (decarboxylating) (...	17	13	5.2	0.00013	0.00013	0.00013	2.5e-05
12	GO:0008948	oxaloacetate decarboxylase activity	17	13	5.2	0.00013	0.00013	0.00013	0.00943
13	GO:0090736	MATH domain binding	10	9	3.06	0.00017	0.00017	0.00017	5.9e-06
14	GO:0004591	oxoglutarate dehydrogenase (succinyl-tra...	18	13	5.51	0.00032	0.00032	0.00032	0.11053
15	GO:0030976	thiamine pyrophosphate binding	18	13	5.51	0.00032	0.00032	0.00032	6.1e-05
16	GO:0008253	5'-nucleotidase activity	9	8	2.75	0.00050	0.00050	0.00050	1.00000
17	GO:0019841	retinol binding	11	9	3.37	0.00067	0.00067	0.00067	0.04032
18	GO:0004494	methylmalonyl-CoA mutase activity	19	13	5.82	0.00074	0.00074	0.00074	0.02899
19	GO:0031419	cobalamin binding	19	13	5.82	0.00074	0.00074	0.00074	0.02784
20	GO:0003777	microtubule motor activity	40	22	12.24	0.00108	0.00108	0.00089	0.00395
21	GO:0019902	phosphatase binding	38	15	11.63	0.15499	0.15499	0.00174	0.00436
22	GO:0004715	non-membrane spanning protein tyrosine k...	25	15	7.65	0.00215	0.00215	0.00215	0.01428
23	GO:0008270	zinc ion binding	186	75	56.93	0.00244	0.00244	0.00244	0.03569
24	GO:0004531	deoxyribonuclease II activity	5	5	1.53	0.00267	0.00267	0.00267	0.04762
25	GO:0030246	carbohydrate binding	86	42	26.32	0.00026	0.00026	0.00390	2.4e-06

26	GO:0004332	fructose-bisphosphate aldolase activity	11	8	3.37	0.00487	0.00487	0.00487	0.33333
27	GO:0004252	serine-type endopeptidase activity	18	11	5.51	0.00705	0.00705	0.00705	0.71571
28	GO:0004657	proline dehydrogenase activity	4	4	1.22	0.00875	0.00875	0.00875	0.13866
29	GO:0016153	urocanate hydratase activity	4	4	1.22	0.00875	0.00875	0.00875	0.00103

BIOLOGICAL PROCESSES

Number	GO:ID	Term	Annotated	Significant	Expected	Classic Fisher	Elim Fisher	Topgo Fisher	Parentchild Fisher
1	GO:0006508	proteolysis	351	172	85.29	6.4e-27	6.4e-27	< 1e-30	2.7e-24
2	GO:0045087	innate immune response	149	105	36.21	< 1e-30	< 1e-30	< 1e-30	2.3e-07
3	GO:0030163	protein catabolic process	232	113	56.38	3.6e-17	3.6e-17	3.5e-21	2.8e-12
4	GO:0070265	necrotic cell death	89	54	21.63	1.3e-13	1.3e-13	1.9e-15	1.7e-08
5	GO:0093002	response to nematicide	23	20	5.59	3.5e-10	3.5e-10	3.5e-10	0.00107
6	GO:0010038	response to metal ion	46	27	11.18	5.5e-07	5.5e-07	6.5e-08	0.00062
7	GO:0006096	glycolytic process	57	32	13.85	2.0e-07	2.0e-07	2.8e-07	0.45241
8	GO:0050829	defense response to Gram-negative bacter...	45	26	10.93	1.4e-06	1.4e-06	1.4e-06	0.06160
9	GO:0045989	positive regulation of striated muscle c...	15	13	3.64	6.0e-07	6.0e-07	2.2e-06	0.00235
10	GO:0008360	regulation of cell shape	35	22	8.5	1.3e-06	1.3e-06	3.3e-06	3.2e-05
11	GO:0051603	proteolysis involved in cellular protein...	123	37	29.89	0.08024	0.08024	6.4e-06	1.00000
12	GO:0060298	positive regulation of sarcomere organiz...	19	14	4.62	7.5e-06	7.5e-06	7.5e-06	2.7e-06
13	GO:0014722	regulation of skeletal muscle contractio...	10	9	2.43	2.2e-05	2.2e-05	2.2e-05	0.00762
14	GO:1905552	positive regulation of protein localizat...	10	9	2.43	2.2e-05	2.2e-05	2.2e-05	2.7e-05
15	GO:0038083	peptidyl-tyrosine autophosphorylation	23	15	5.59	3.5e-05	3.5e-05	3.5e-05	0.00702
16	GO:0008152	metabolic process	2159	521	524.64	0.64741	1.00000	5.7e-05	0.64741
17	GO:0030317	flagellated sperm motility	13	10	3.16	9.4e-05	9.4e-05	9.4e-05	0.28571
18	GO:0035023	regulation of Rho protein signal transdu...	11	9	2.67	9.6e-05	9.6e-05	9.6e-05	0.01300
19	GO:0050790	regulation of catalytic activity	96	21	23.33	0.74947	0.74947	0.00011	0.36741
20	GO:0040002	collagen and cuticulin-based cuticle dev...	33	21	8.02	1.6e-06	0.00015	0.00015	0.04552
21	GO:0007018	microtubule-based movement	69	31	16.77	0.00012	0.01642	0.00017	0.00102
22	GO:0051046	regulation of secretion	33	10	8.02	0.26582	0.26582	0.00021	0.26770
23	GO:1905905	pharyngeal gland morphogenesis	16	11	3.89	0.00021	0.00021	0.00021	0.79340

24	GO:0040018	positive regulation of multicellular org...	39	20	9.48	0.00023	0.00023	0.00023	0.03389
25	GO:0019556	histidine catabolic process to glutamate...	8	7	1.94	0.00031	0.00031	0.00031	0.02249
26	GO:0019557	histidine catabolic process to glutamate...	8	7	1.94	0.00031	0.00031	0.00031	0.02249
27	GO:0030048	actin filament-based movement	13	8	3.16	0.00460	0.00460	0.00031	0.03626
28	GO:0010172	embryonic body morphogenesis	49	23	11.91	0.00042	0.00042	0.00042	0.00020
29	GO:0030241	skeletal muscle myosin thick filament as...	15	10	3.64	0.00060	0.00060	0.00060	0.03251
30	GO:0007155	cell adhesion	92	38	22.36	0.00019	0.00019	0.00099	4.3e-06
31	GO:1902075	cellular response to salt	9	7	2.19	0.00110	0.00110	0.00110	0.00180
32	GO:0019511	peptidyl-proline hydroxylation	10	8	2.43	0.00033	0.00033	0.00111	0.00478
33	GO:0032456	endocytic recycling	12	8	2.92	0.00224	0.00224	0.00112	0.00015
34	GO:0007032	endosome organization	8	7	1.94	0.00031	0.00031	0.00112	0.00100
35	GO:0007060	male meiosis chromosome segregation	9	7	2.19	0.00110	0.00110	0.00112	7.1e-05
36	GO:0007605	sensory perception of sound	7	6	1.7	0.00113	0.00113	0.00113	0.03170
37	GO:0031272	regulation of pseudopodium assembly	7	6	1.7	0.00113	0.00113	0.00113	0.01457
38	GO:0030330	DNA damage response, signal transduction...	16	8	3.89	0.02269	0.02269	0.00113	0.01190
39	GO:0040017	positive regulation of locomotion	84	29	20.41	0.02123	0.02123	0.00191	0.01055
40	GO:0016477	cell migration	143	42	34.75	0.09067	0.09067	0.00209	0.99789
41	GO:0018105	peptidyl-serine phosphorylation	54	23	13.12	0.00218	0.00218	0.00218	0.09726
42	GO:0009117	nucleotide metabolic process	165	46	40.09	0.15665	0.99846	0.00222	1.00000
43	GO:2000057	negative regulation of Wnt signaling pat...	12	8	2.92	0.00224	0.00224	0.00224	0.00177
44	GO:0042338	cuticle development involved in collagen...	15	9	3.64	0.00333	0.00333	0.00333	0.32661
45	GO:0045732	positive regulation of protein catabolic...	18	6	4.37	0.25831	0.25831	0.00349	0.76718

46	GO:0009258	10-formyltetrahydrofolate catabolic proc...	6	5	1.46	0.00402	0.00402	0.00402	0.08392
47	GO:0010133	proline catabolic process to glutamate	6	5	1.46	0.00402	0.00402	0.00402	0.09904
48	GO:0019563	glycerol catabolic process	6	5	1.46	0.00402	0.00402	0.00402	1.00000
49	GO:0045887	positive regulation of synaptic assembly...	16	9	3.89	0.00599	0.00599	0.00599	0.41315
50	GO:0050832	defense response to fungus	15	8	3.64	0.01434	0.01434	0.00630	0.82414
