# Supplementary material for: Assembly and Analysis of Haemonchus contortus Transcriptome as a Tool for the Knowledge of Ivermectin Resistance Mechanisms
Source: Pathogens. 2023 Mar 22;12(3):499. doi: 10.3390/pathogens12030499 (PMC10059914; doi:10.3390/pathogens12030499)
Supplement: Supplementary file 1 [file pathogens-12-00499-s001.zip › S6_Upregulated GO terms in IVMs LFC2.pdf]

UPREGULATED ENRICHED GENE ONTOLOGY TERMS PER CATEGORY IN *Haemonchus contortus* IVERMECTIN SUSCEPTIBLE STRAIN (IVMs), USING LFC ≥ 2 (p value ≤ 0.01)

CELLULAR COMPONENTS

Number	GO:ID	Term	Annotated	Significant	Expected	Classic Fisher	Elim Fisher	Topgo Fisher	Parentchild Fisher
1	GO:0032040	small-subunit processome	16	11	3.06	2.0e-05	2.0e-05	2.0e-05	0.01314
2	GO:0005840	ribosome	135	50	25.83	5.2e-07	5.9e-05	5.9e-05	1.5e-05
3	GO:0042555	MCM complex	7	6	1.34	0.00028	0.00028	0.00028	0.00068
4	GO:0005743	mitochondrial inner membrane	150	43	28.7	0.00252	0.00252	0.00122	0.15066
5	GO:0071011	precatalytic spliceosome	22	10	4.21	0.00429	0.00429	0.00429	0.08152
6	GO:0005634	nucleus	1022	252	195.54	1.1e-07	0.00047	0.00548	6.5e-07
7	GO:0097526	spliceosomal tri-snRNP complex	15	7	2.87	0.01403	0.01403	0.00561	0.02249
8	GO:0005687	U4 snRNP	5	4	0.96	0.00564	0.00564	0.00564	0.01285
9	GO:0016342	catenin complex	5	4	0.96	0.00564	0.00564	0.00564	0.00167
10	GO:0042788	polysomal ribosome	5	4	0.96	0.00564	0.00564	0.00564	0.06382

MOLECULAR FUNCTIONS

Number	GO:ID	Term	Annotated	Significant	Expected	Classic Fisher	Elim Fisher	Topgo Fisher	Parentchild Fisher
1	GO:0045735	nutrient reservoir activity	19	19	3.46	6.9e-15	6.9e-15	6.9e-15	6.9e-15
2	GO:0005319	lipid transporter activity	26	20	4.73	9.3e-11	9.3e-11	1.1e-10	2.8e-09
3	GO:0004360	glutamine-fructose-6-phosphatetransamin...	8	8	1.45	1.2e-06	1.2e-06	1.2e-06	1.00000
4	GO:0003735	structural constituent of ribosome	118	41	21.46	9.5e-06	9.5e-06	9.5e-06	0.02726
5	GO:0003730	mRNA 3'-UTR binding	16	10	2.91	0.00010	0.0001	0.00010	0.04411
6	GO:0003677	DNA binding	218	60	39.64	0.00029	0.0010	0.00048	0.72431
7	GO:0004617	phosphoglycerate dehydrogenase activity	4	4	0.73	0.00109	0.0011	0.00109	0.00071
8	GO:0004129	cytochrome-c oxidase activity	35	14	6.36	0.00200	0.0020	0.00200	0.09351
9	GO:0004693	cyclin-dependent protein serine/threonin...	15	8	2.73	0.00221	0.0022	0.00221	0.00011
10	GO:0005109	frizzled binding	5	4	0.91	0.00464	0.0046	0.00464	0.04545
11	GO:0035198	miRNA binding	5	4	0.91	0.00464	0.0046	0.00464	0.33333
12	GO:0045294	alpha-catenin binding	5	4	0.91	0.00464	0.0046	0.00464	0.00289
13	GO:0003688	DNA replication origin binding	3	3	0.55	0.00599	0.0060	0.00599	0.01833
14	GO:0003987	acetate-CoA ligase activity	3	3	0.55	0.00599	0.0060	0.00599	0.25000
15	GO:0004638	phosphoribosylaminoimidazole carboxylase...	3	3	0.55	0.00599	0.0060	0.00599	0.00119
16	GO:0004639	phosphoribosylaminoimidazolesuccinocarbo...	3	3	0.55	0.00599	0.0060	0.00599	0.05000
17	GO:0008061	chitin binding	3	3	0.55	0.00599	0.0060	0.00599	0.00472
18	GO:0016208	AMP binding	3	3	0.55	0.00599	0.0060	0.00599	0.00423
19	GO:0017147	Wnt-protein binding	3	3	0.55	0.00599	0.0060	0.00599	0.00417
20	GO:0035242	protein-arginine omega-N asymmetric meth...	3	3	0.55	0.00599	0.0060	0.00599	0.25000
21	GO:0004748	ribonucleoside-diphosphate reductase act...	8	5	1.45	0.00676	0.0068	0.00676	1.00000
22	GO:0009055	electron transfer activity	81	29	14.73	0.00011	0.0116	0.00786	0.00032

BIOLOGICAL PROCESSES

Number	GO:ID	Term	Annotated	Significant	Expected	Classic Fisher	Elim Fisher	Topgo Fisher	Parentchild Fisher
1	GO:0006048	UDP-N-acetylglucosamine biosynthetic pro...	12	11	2.39	1.8e-07	1.8e-07	1.8e-07	0.00898
2	GO:0006541	glutamine metabolic process	20	13	3.98	1.3e-05	1.3e-05	4.4e-05	0.00014
3	GO:0006412	translation	211	63	42.04	0.00023	0.00080	6.2e-05	0.02089
4	GO:0006002	fructose 6-phosphate metabolic process	10	8	1.99	7.3e-05	7.3e-05	7.3e-05	0.00012
5	GO:0006189	'de novo' IMP biosynthetic process	8	7	1.59	8.0e-05	8.0e-05	8.0e-05	0.06667
6	GO:0090727	positive regulation of brood size	8	7	1.59	8.0e-05	8.0e-05	8.0e-05	0.06667
7	GO:0006260	DNA replication	62	34	12.35	8.1e-10	9.0e-05	9.7e-05	5e-06
8	GO:0006564	L-serine biosynthetic process	5	5	1	0.00031	0.00031	0.00031	0.01299
9	GO:0048557	embryonic digestive tract morphogenesis	17	10	3.39	0.00046	0.00046	0.00046	0.00523
10	GO:0000470	maturation of LSU-rRNA	19	9	3.79	0.00632	0.00632	0.00055	0.29550
11	GO:0006364	rRNA processing	77	32	15.34	9.0e-06	0.01625	0.00187	0.11049
12	GO:0007049	cell cycle	344	88	68.53	0.00399	0.05548	0.00208	0.01212
13	GO:0042074	cell migration involved in gastrulation	14	8	2.79	0.00228	0.00228	0.00228	0.01355
14	GO:0006355	regulation of transcription, DNA-templat...	253	60	50.4	0.06963	0.06963	0.00240	0.53993
15	GO:0007017	microtubule-based process	192	37	38.25	0.62242	0.62242	0.00472	0.72824
16	GO:0002159	desmosome assembly	5	4	1	0.00658	0.00658	0.00658	0.03186
17	GO:0006069	ethanol oxidation	5	4	1	0.00658	0.00658	0.00658	0.01374
18	GO:0090138	regulation of actin cytoskeleton organiz...	5	4	1	0.00658	0.00658	0.00658	0.00059
19	GO:0006552	leucine catabolic process	3	3	0.6	0.00788	0.00788	0.00788	0.00149
20	GO:0019413	acetate biosynthetic process	3	3	0.6	0.00788	0.00788	0.00788	0.00075
21	GO:0019427	acetyl-CoA biosynthetic process from ace...	3	3	0.6	0.00788	0.00788	0.00788	0.25000
22	GO:0019542	propionate biosynthetic process	3	3	0.6	0.00788	0.00788	0.00788	1.00000
23	GO:0034247	snoRNA splicing	3	3	0.6	0.00788	0.00788	0.00788	0.01906
24	GO:0043054	dauer exit	3	3	0.6	0.00788	0.00788	0.00788	0.00681
25	GO:0048146	positive regulation of fibroblast prolif...	3	3	0.6	0.00788	0.00788	0.00788	0.01422
26	GO:0061158	3'-UTR-mediated mRNA destabilization	3	3	0.6	0.00788	0.00788	0.00788	0.04396
27	GO:0090038	negative regulation of protein kinase C ...	3	3	0.6	0.00788	0.00788	0.00788	0.02299
28	GO:2000114	regulation of establishment of cell pola...	3	3	0.6	0.00788	0.00788	0.00788	0.01140
